# CMR validation of fractional changes in annulo-apical angles and TAPSE for rapid assessment of right ventricular systolic function

**DOI:** 10.1186/1532-429X-14-S1-P284

**Published:** 2012-02-01

**Authors:** Simon A Zakeri, Alexander Borg, Matthias Schmitt

**Affiliations:** 1 University of Manchester, Manchester, UK; 2 Cardiology, Cardiovascular Division, University Hospital of South Manchester, Manchester, UK; 3 Manchester Medical School, Manchester, UK

## Background

Volumetric assessment of the right ventricle (RV) by Cardiac Magnetic Resonance (CMR), albeit time-consuming, provides accurate and reproducible measurement of RV ejection fraction (RVEF). Tricuspid annulus peak systolic excursion (TAPSE) is a predominantly Echo-validated rapidly-derived surrogate of RV function. Correlations between RVEF and systolic changes in annulo-apical angles (AAAs) have not previously been evaluated.

## Objective

To assess the use of changes in AAAs and TAPSE as rapidly-derived surrogate markers of RV systolic function using CMR.

## Methods

**Figure 1:**
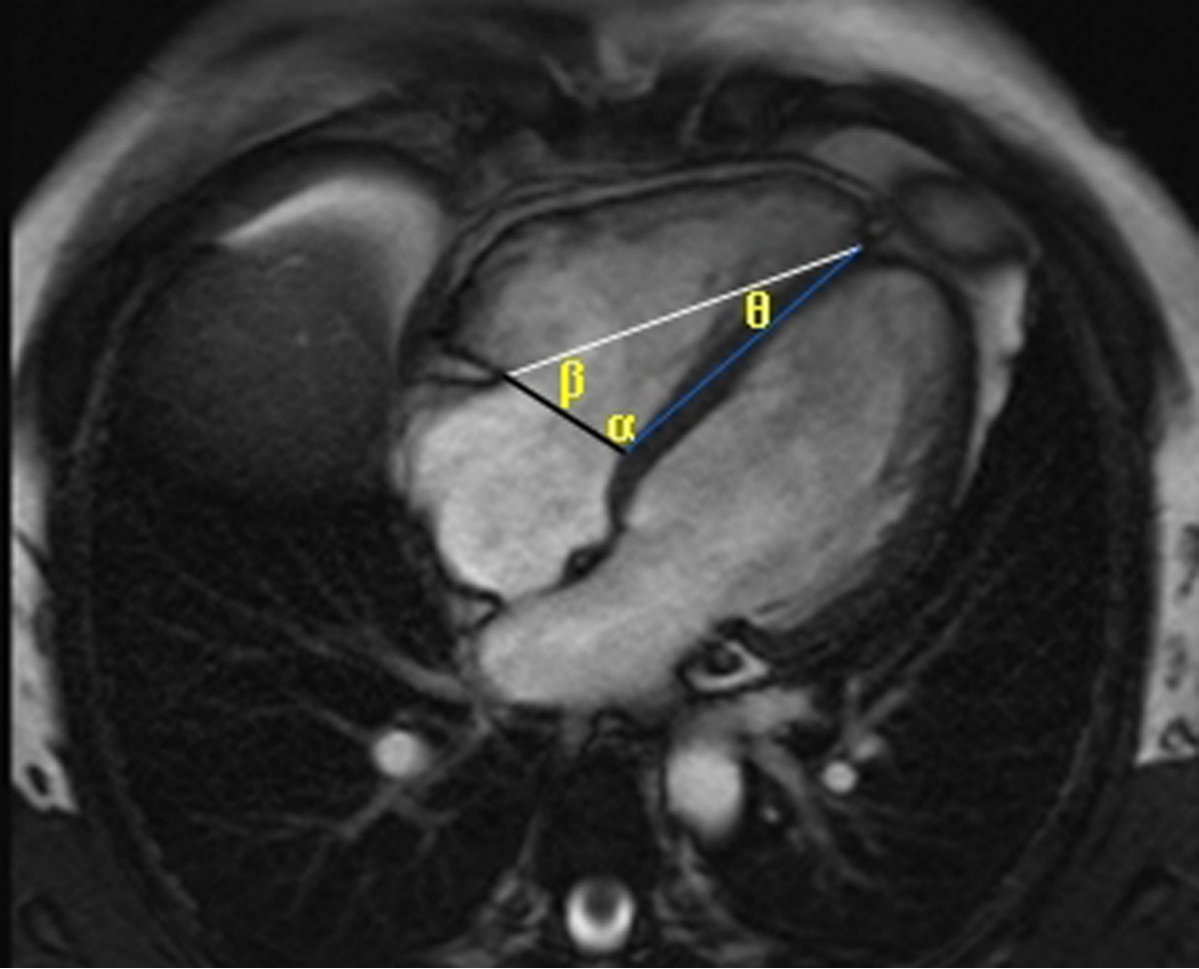
AAAs in ED on a 4chamber view.

We measured RV volumes from short-axis bSSFP stacks in patients undergoing clinically indicated CMR scans. RVEF was calculated from volumes derived by semi-automated endocardial contouring (QMass®MR 7.2). AAAs (α,β,θ angles -see figure 1), subtended by a triangle connecting the medial and lateral extent of the tricuspid valve annulus and RV apex, and fractional changes in AAAs (ΔAAA/EDAAAx100, whereby ΔAAA=EDAAA-ESAAA) were measured from end-diastolic (ED) and end-systolic (ES) 4chamber SSFP cine still frames. TAPSE was measured as the change in length of a line connecting the lateral tricuspid valve annulus with the RV apex from ED to ES. Parameters were compared with RVEF using Spearman rank correlations; ROC curves constructed to assess accuracy of the parameters in predicting an RVEF<50%.

**Figure 2:**
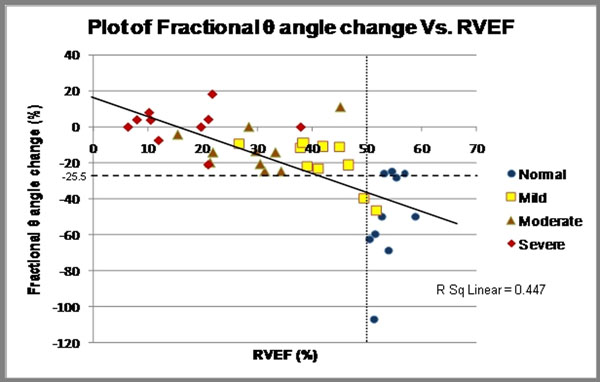
Scatter graph -fractional θ angle change vs. RVEF, with line of best fit. Patient subgroups are colour coded. The dotted vertical line represents the ROC cut-off of RVEF<50%, and the dashed horizontal line represents the corresponding cut-off value for fractional θ angle change of ≥ -25.5%.

## Results

Forty subjects were included: 10 normals, 10 mildly-impaired, 10 moderately-impaired, and 10 with severely-impaired RV systolic function. Median (25th-75th percentile) RVEF for each subgroup was 53.5% (51.4%-55.7%), 41.5% (38.1%-47.2%), 30.0% (21.7%-33.5%), and 15.8% (9.6%-21.2%), respectively. Correlations with RVEF: TAPSE (0.74 p<0.001), fractional changes of α angle (0.64, p<0.001), β angle (-0.39, p<0.05), and θ angle, which had the highest correlation (-0.77, p<0.001). Smaller increases or a decrease in magnitude of the θ angle from ED to ES are associated with lower RVEFs, whereby a fractional θ angle change of ≥ -25.5% predicts RVEF<50% [97% sensitivity, 91% specificity, AUC=0.98]. The cut-off for TAPSE is ≤1.87cm [100% sensitivity, 82% specificity, AUC=0.98]. Intra- and inter-observer reproducibility is excellent as shown by intra-class correlation coefficients for TAPSE (0.98 and 0.92, respectively) and fractional θ angle change (0.96 and 0.80, respectively).

## Conclusions

Both fractional θ angle change and TAPSE strongly correlate with RVEF, and are accurate predictors of RVEF<50%. These measurements provide an excellent alternative to the more time-consuming derivation of RVEF obtained volumetrically by endocardial chamber tracing.

## Funding

No funding.

